# A comparative metabolomic study on desi and kabuli chickpea (*Cicer arietinum* L.) genotypes under rainfed and irrigated field conditions

**DOI:** 10.1038/s41598-020-70963-6

**Published:** 2020-08-18

**Authors:** Zaib Un Nisa, Anjuman Arif, Muhammad Qandeel Waheed, Tariq Mahmood Shah, Ayesha Iqbal, Amna Jabbar Siddiqui, Muhammad Iqbal Choudhary, Hesham R. El-Seedi, Syed Ghulam Musharraf

**Affiliations:** 1 H.E.J. Research Institute of Chemistry, International Center for Chemical and Biological Sciences, University of Karachi, Karachi, 75270 Pakistan; 2 Dr. Panjwani Center for Molecular Medicine and Drug Research, International Center for Chemical and Biological Sciences, University of Karachi, Karachi, 75270 Pakistan; 3 Nuclear Institute for Agriculture & Biology (NIAB), Faisalabad, 38000 Pakistan; 4 Department of Biochemistry, Faculty of Science, King Abdulaziz University, Jeddah, 21589 Saudi Arabia; 5 Pharmacognosoy Group, Department of Medicinal Chemistry, Biomedical Centre, Uppsala University, Box 574, 75 123 Uppsala, Sweden; 6 International Research Center for Food Nutrition and Safety, Jiangsu University, Zhenjiang, 212013 China

**Keywords:** Plant sciences, Chemistry

## Abstract

Chickpea is considered among the most important leguminous crops in the world. However, in recent years drought conditions and/or limited availability of water have significantly reduced the production of chickpea. The current study was aimed to understand the legume stress response at the metabolic level for the determination of chickpea genotypes which can resist yield losses and could be cultivated with limited water availability. Here, we have analyzed two genotypes of chickpea, desi and kabuli under rainfed condition using a GC–MS based untargeted metabolomics approach. Results revealed significant differences in several metabolite features including oxalic acid, threonic acid, inositol, maltose and l-proline between studied groups. Accumulation of plant osmoprotectants such as l-proline, sugars and sugar alcohols was higher in desi genotype than kabuli genotype of chickpea when grown under the rainfed condition. Metabolic pathway analysis suggests that the inositol phosphate metabolism was involved in plant defense mechanisms against the limited water availability.

## Introduction

Chickpea (*Cicer arietinum* L.), the third most important legume crop of the world, is grown in nearly 52 countries^1^. South Asia is the leading producer of chickpea which contributes about to three-quarters of the global chickpea production^2^. There are two main types of cultivated chickpea genotypes, desi and kabuli which can be distinguished by the size, shape and color of the seeds. Kabuli genotypes have larger, rounder and cream-colored seeds that are largely grown in North Africa, West Asia, North America and Europe, whereas desi genotypes have relatively smaller, angular-shaped and dark colored seeds mostly grown in Asia and Africa^3^. Chickpea seeds have good nutritional value—they contain high amounts of unsaturated fatty acids and are an inexpensive source of high quality plant protein for millions of people in developing countries^4,5^. They are also rich in minerals, dietary fibers and vitamins such as tocopherol (both γ and α), folic acid, riboflavin (B2), pantothenic acid (B5), pyridoxine (B6), and carotenoids such as β-carotene, lutein, cryptoxanthin and zeaxanthin^6^.

Chickpea plants have a deep taproot system which helps them extract water from deeper soil layers and enhances their capacity to withstand limited water stress. Chickpea is a crop of temperate areas and most of its cultivation is done on the sandy loam soils under low-rainfall conditions. Loam and fertile sandy soil have good internal drainage; therefore they are considered the best mediums for the growth of chickpea plants^2,7^. In the rainfed areas, chickpea production is low and unreliable due to these marginal lands where the success of crops is dependent upon the availability of soil moisture through rains.

In Pakistan, chickpea is cultivated in rainfed areas i.e. sandy desert, which is not connected to the irrigation system. Chickpea is the only crop that can be cultivated due to its deep taproot system which helps it survive in desert lands where no other crop plant can survive. Chickpea produces more grains under limited water conditions because with excessive rain/irrigation chickpea growth becomes inclined towards producing more leaves than pods.

Our main focus of this study was to assess the drought stress responses of kabuli and desi chickpea genotypes when grown in chickpea cultivation rainfed areas as opposed to in the greenhouse. This strategy was based on the understanding that unlike cereals, legumes cannot be grown well in greenhouses or in pots, an artificial environment where all conditions like temperature, irrigation and light can be controlled. Moreover, plants skip facing natural environmental factors like biotic and abiotic stresses when grown in greenhouses. Due to unpredictable climate change, climate-resilient crops must be developed with the ability to grow in natural farmer fields with resistance to stresses present in these open fields. In the present study, we adopted this approach to evaluate advanced breeding lines of chickpea in open fields. Drought stress was intended to be induced by growing chickpea in one of such areas; Kallur Kot, situated in desert Thal, District Bhakkar in Punjab. Nuclear Institute for Agriculture and Biology (NIAB) farms were used as irrigated controls that are located in Faisalabad division. The Faisalabad division shares some environmental factors with the selected rainfed area but differs in soil structure, composition and rainfall intensity. A description of the environment is given in supplementary information (Table S1). The yield was taken as the determining factor of drought tolerance in this study.

Plants decrease stomatal conductance during water scarcity which results in a reduced CO_2_ fixation, and a decrease in the rate of photosynthesis, followed by a reduction in growth and yield of plants^8,9^. However, plants can protect themselves against mild drought stress by accumulating osmolytes^10^. Osmolytes are low-molecular weight organic compounds which are also known as compatible solutes because they are non-toxic for cells and do not interfere with normal metabolism, even when present at higher concentrations^11^. Targeting osmolytes and other small molecules in order to study legume stress response could help in the understanding of the mechanisms that plants adapt to maintain their homeostasis under abiotic stress conditions. To achieve this goal, we focus on metabolomics, which plays an important role in understanding the complex shifts that occur in plants under environmental perturbation, such as drought and/or limited water stress, salinity, nutrient limitation, extreme temperature as well as biotic stresses, such as pathogen attacks^12^. Influence of water stress on chlorophyll content, proline content, transpiration, photosynthesis and stomatal conductance has been reported in chickpea in drought-tolerant and drought-sensitive varieties^13^. Also, photosynthetic characteristics, leaf water potential, and reproductive development of two chickpea genotypes with different yields in the field have been compared and reported under terminal drought stress^8^. Recently, variations in metabolic levels were reported in drought-sensitive and tolerant varieties of chickpea which were grown in a greenhouse^14^.

In this study, untargeted metabolite profiling of advanced breeding lines of desi and kabuli chickpea genotypes was performed in order to identify the metabolites associated with drought-tolerant genotype of chickpea. The results of this study can further be used to predict gene functions by analyzing associated metabolites.

## Results

### Measurements of drought tolerance of chickpea genotypes

In the present study, the drought tolerance of chickpea genotypes was measured as a function of yield performance in control (irrigated) and drought (rainfed) stressed conditions. Plants grown in rainfed desert areas were assumed to experience drought stress mainly due to (i) limited rainfall and (ii) soil composition and structure in comparison to the irrigated control where the soil is more fertile and which has relatively more rainfall. However, among chickpea genotypes, desi type is reported to be more tolerant to drought stress than the kabuli genotypes^15,16^. Farooq et al., reported that desi genotypes were found more tolerant to drought stress due to greater accumulation of osmolytes than kabuli genotypes^17^.

Our results also validate these findings and revealed that the kabuli genotypes displayed relatively greater yield reduction when subjected to drought stress under natural field conditions in comparison to their respective irrigated controls as compared to desi genotypes (Table 1; Supplementary Material, Fig. 1).

**Table 1:** Mean grain yields of genotype groups in control (irrigated) and drought (rainfed) conditions.

Sr. no.	Genotype	Type	Grain yield (g)/plot^a^		Yield loss in drought (%)^b^
			Control	Drought	
1	CH 40/09	Desi	819	536	35
2	CH 39/08	Desi	928	808	13
3	DCD	Desi	1,192	344	71
4	CH 49/09	Desi	728	464	36
5	AZC	Desi	736	360	51
6	D-13036	Desi	817	544	33
7	D-13012	Desi	958	416	57
8	D-13011	Desi	938	400	57
9	CH 32/10	Desi	794	440	45
10	D-13029	Desi	770	456	41
11	D-13031	Desi	799	400	50
12	CM584/09	Desi	792	472	40
13	CH10/08	Desi	792	648	18
14	CH 1/11	Desi	704	512	27
15	CH 3/11	Desi	641	336	48
16	CH 13/11	Desi	752	488	35
17	CH 50/11	Desi	672	272	60
18	CH28/10	Desi	840	496	41
19	PB-2000	Desi	688	384	44
20	Bittel-16	Desi	720	328	54
21	CH-2016	Desi	720	467	35
22	Paidar-91	Desi	400	224	44
23	E-26	Desi	498	352	29
24	K-850	Desi	640	616	4
25	BKK 2,174	Desi	1,009	512	49
26	BK-2011	Desi	1,026	352	66
		Mean	784	447	42
1	K-01216	Kabuli	848	190	78
2	CH 55/09	Kabuli	944	224	76
3	K-01211	Kabuli	1,031	312	70
4	CH 61/09	Kabuli	760	344	55
5	TG12K-07	Kabuli	881	440	50
6	BKK2174	Kabuli	1,056	472	55
7	CH76/08	Kabuli	765	424	45
8	CH 77/08	Kabuli	968	288	70
9	K-01209	Kabuli	691	408	41
10	DG-2017	Kabuli	439	130	70
11	K-01241	Kabuli	898	330	63
12	K-01308	Kabuli	863	384	56
13	K-01242	Kabuli	601	352	41
14	CM877/10	Kabuli	624	424	32
15	K-01240	Kabuli	621	144	77
16	TG12K02	Kabuli	501	160	68
17	Noor-13	Kabuli	792	344	57
18	CM2008	Kabuli	770	376	51
		Mean	781	319	59

^a^Mean grain Yield (g) per plot (1.8 m^2^).

^b^Mean yield loss in drought as percentage of control mean yield.

Besides, analysis of variance also showed that variations in grain yield caused by drought stress in Kabuli genotypes were more pronounced in approximately 73% of total variations; however, the figure was 58% in desi genotypes. Similarly, genotype variations in kabuli genotypes were lesser—i.e. 19% of total variations—than desi genotypes i.e. 26% (Table 2), which indicates that Desi chickpea has more diversity in it when grain yield was accounted for in stressed and non-stressed conditions. These results clearly indicate that desi genotypes acquired better drought tolerance from mother nature than kabuli genotypes.

**Table 2:** Analysis of variance grain yield vs treatment, genotypes.

Source	DF	SS (%)	MS	F	P
**Desi-genotypes**					
Genotype	25	658,331 (26)	26,333	1.67	0.104 ns
Treat	1	1,471,010 (58)	1,471,010	93.24	0**
Error	25	394,397 (16)	15,776		
Total	51	2,523,738 (100)			
**Kabuli-genotypes**					
Genotype	17	492,220 (19)	28,954	2.18	0.059 ns
Treat	1	1,916,840 (73)	1,916,840	144.41	0**
Error	17	225,652 (9)	13,274		
Total	35	2,634,713 (100)			

### Metabolite analysis of desi type of chickpea

After alignment of the data of desi chickpea plants, a total of 414 metabolites were detected and filtered by frequency, fold change and probability. T-test was applied to determine the significant difference between rainfed and irrigated control samples at probability 0.05 and fold change > 1.5 and a list of 19 metabolites was found to be significantly different on these parameters. Among them, 11 compounds were found to be up-regulated and 8 were down-regulated (Table 3). Up-regulated metabolites were including sugars; d-fructose, allose, α -d-glucopyranoside, and fucose; two sugar alcohols inositol, myo-inositol; and other compounds such as malic acid, l-proline, ethylamine, butane 1,2,3-triol. While four acids including threonic acid, gluconic acid, malonic acid, oxalic acid; two sugar alcohols including xylitol, erythritol; and a sugar arabinofuranose were down-regulated.

**Table 3:** List of up-and down-regulated metabolites in desi chickpea genotypes grown under rainfed conditions in comparison to irrigated controls.

S. no.	Compound (CAS registry numbers)	Retention time	*p* (corr) rainfed vs control	Log FC (abs) rainfed vs control
**List of up-regulated metabolites**				
1	α-d-Glucopyranoside (19159-25-2)	43.79	3.63E−08	1.36
2	d-Fructose (19126-98-9)	29.20	5.01E−04	0.93
3	Inositol (29412-27)	32.00	9.83E−15	1.68
4	Malic acid (38166-11-9)	21.60	6.74E−13	1.62
5	Butane 1,2,3-triol (33581-76-9)	16.50	2.38E−07	1.33
6	73.0*	22.79	8.12E−06	1.15
7	Myo-Inositol (2582-79-8)	34.50	3.67E−21	1.82
8	l-Proline (7364-47-8)	16.30	4.50E−06	1.18
9	Allose (2595-97-3)	30.10	4.50E−06	1.18
10	Fucose (117307-13-8)	34.20	1.21E−06	1.24
11	Ethylamine (2477-39-6)	5.299	4.50E−06	1.18
**List of down-regulated metabolites**				
1	Threonic acid (13752-84-6)	23.50	8.29E−15	− 1.69
2	Xylitol (14199-72-5)	26.62	1.90E−17	− 1.75
3	Malonic acid (18457-04-0)	13.60	3.50E−10	− 1.49
4	Erythritol (18547-29-0)	22.79	3.83E−06	− 1.19
5	Arabinofuranose (43225-70-3)	28.26	1.22E−05	− 1.13
6	Oxalic acid (18294-04-7)	19.20	1.97E−07	− 1.31
7	306.0*	30.70	2.33E−08	− 1.38
8	Gluonic acid (34290-52-3 )	30.50	6.01E−13	− 1.62

*m/z of base peak of unidentified metabolite.

**Figure 1:**
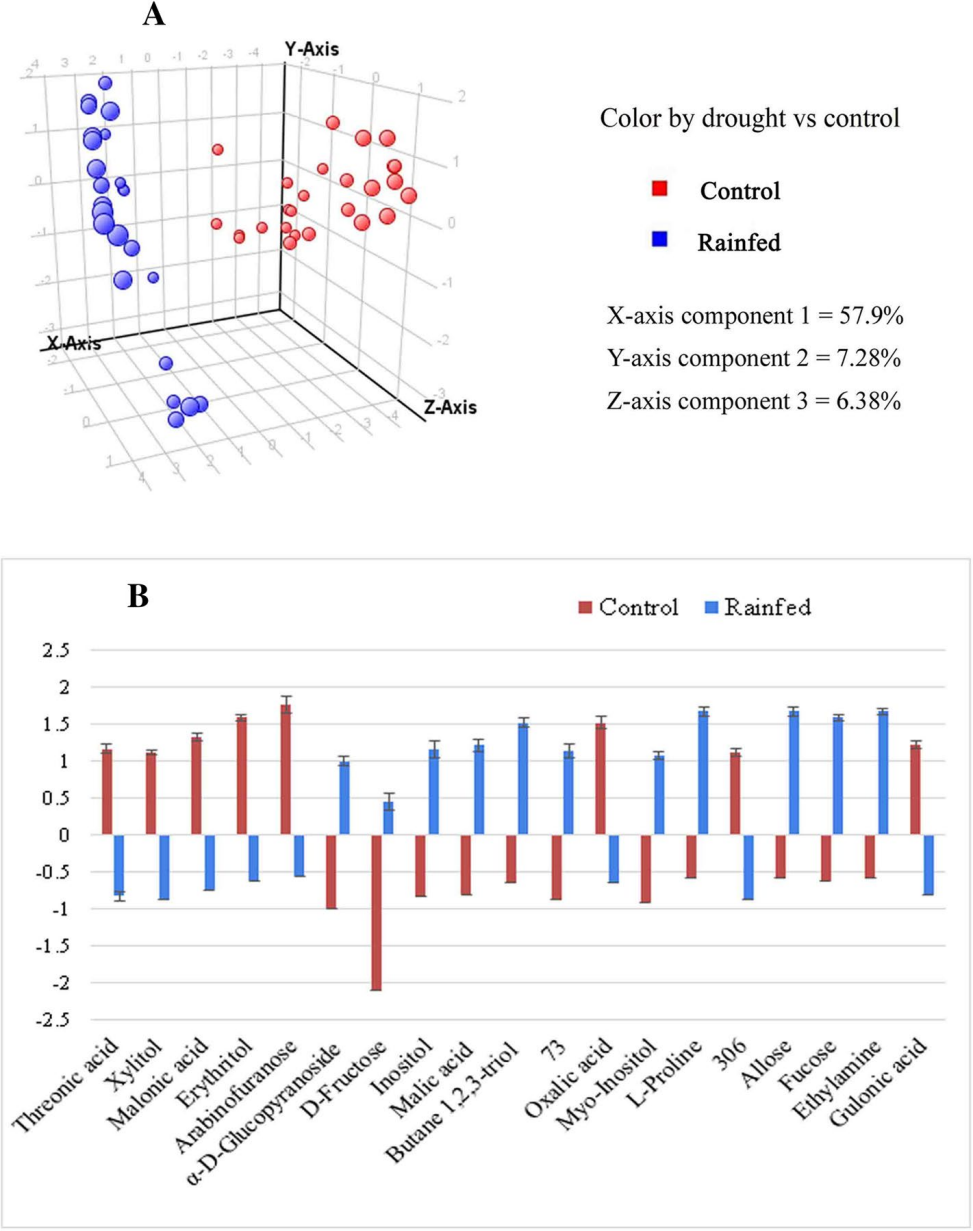
(**A**) PCA score plot of control and rainfed samples of desi chickpea genotypes. (**B**) Bar chart showing up-and down-regulation of 19 significant metabolites under rainfed condition in comparison to irrigated controls, i.e. at fold change > 1.5. (11 metabolites are upregulated and 8 are down regulated).

PCA was generated which showed a notable trend of separation for the two groups i.e. the irrigated control plants and the plants grown under rainfed conditions (Fig. 1A). Each sample in this PCA score is represented by a single point. The variance of the first three components on X, Y, and Z were found to be 57.9%, 7.28%, and 6.37%, respectively. The bar chart was drawn on the basis of normalized average intensities of 19 metabolites showing an up-regulation of 11 metabolites (bar above the baseline of 0) and downregulation of 8 metabolites (bar below the baseline of 0) in rainfed samples as compared to irrigated control (Fig. 1B).

**Figure 2:**
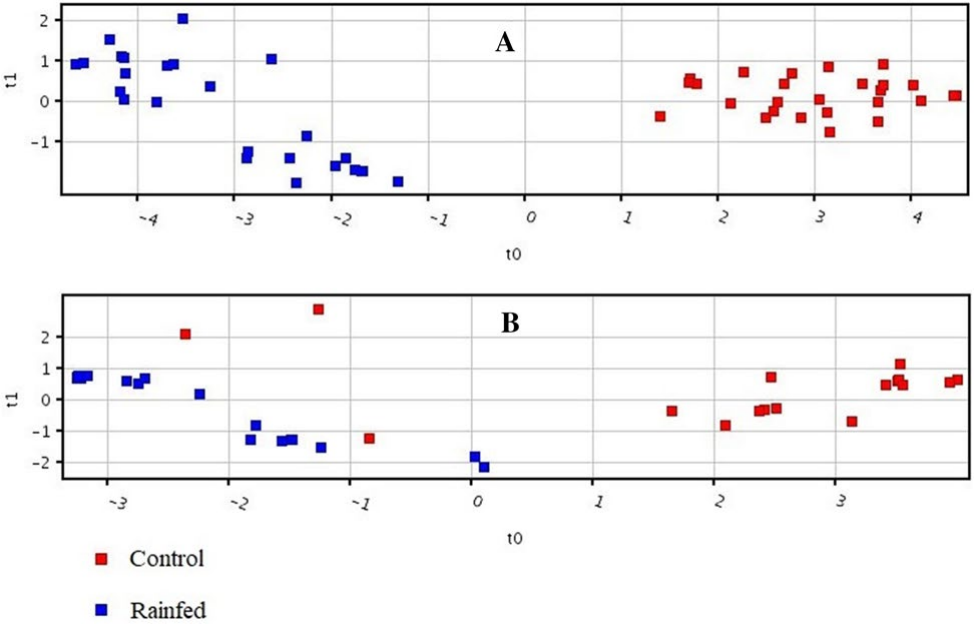
PLS-DA score scatter plots of chickpea genotypes discriminating among rainfed and irrigated (control) plants based on their differentiated metabolites data, (**A**) desi genotypes, (**B**) kabuli genotypes.

Supervised Partial Least Square Discriminant Analysis (PLS-DA) was performed and a model was built in order to classify samples into discrete classes. The dataset was divided into two equal groups: one part was used for training, and the other part for testing. Thus, a confusion matrix was generated. The results of the confusion matrix are provided in the supplementary information (Table S2). Plot obtained by PLS-DA score (Fig. 2A), showing a clear separation trend between the rainfed conditions and irrigated control plants. Sensitivity and specificity were also measured from the constructed model, and was found to be 100%.

### Metabolite analysis of kabuli type of chickpea

**Figure 3:**
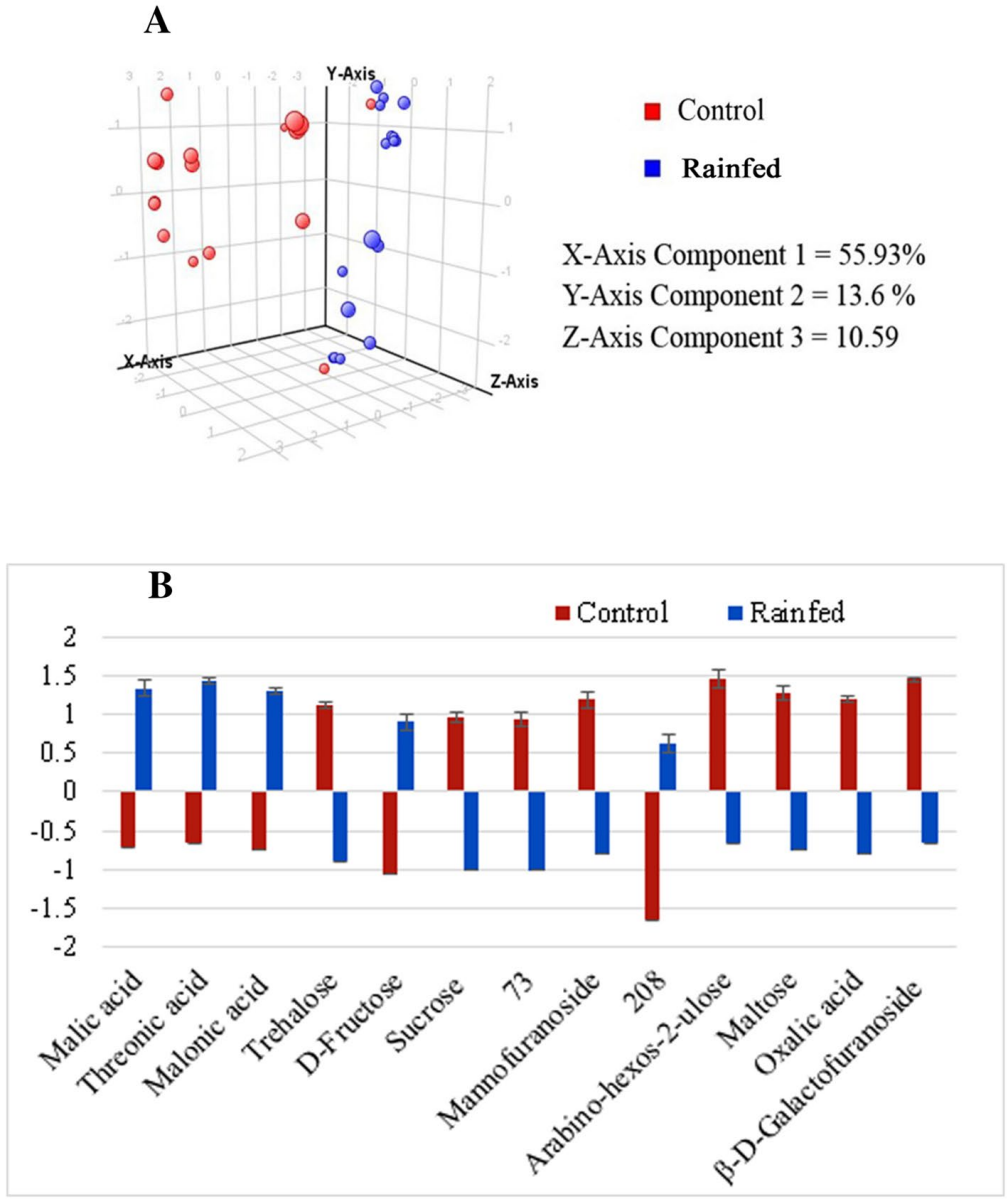
(**A**) PCA score plot of control and rainfed samples of kabuli chickpea genotypes. (**B**) Bar chart showing up-and down-regulation of 13 significant metabolites under rainfed condition in comparison to irrigated controls, i.e. at fold change > 1.5. (5 metabolites are upregulated and 8 are down regulated).

A total of 260 metabolites were detected from the kabuli chickpea samples and a list of 13 metabolites was found to be significantly different between rainfed and irrigated control group using t-test at the probability of 0.05 and fold change > 1.5. Mannofuranoside, arabino-hexose-2-ulose, maltose, beta-D-glactofuranoside, sucrose, trehalose and oxalic acid were found to be down-regulated, while three organic acids, malic acid, threonic acid, malonic acid, and a sugar d-fructose were up-regulated (Table 4). PCA model was generated on kabuli chickpea samples, which showed a notable difference between irrigated control plants and the plants grown under rainfed conditions. The variance of the first three components on X, Y, and Z were found to be 55.93%, 13.6%, and 10.95%, respectively, (Fig. 3A). The bar chart was built on the basis of normalized average intensities of 13 metabolites, showing an up-regulation of 5 metabolites (bar above the baseline of 0) and downregulation of 8 metabolites (bar below the baseline of 0) in rainfed samples as compared to irrigated control as shown in Fig. 3B.

**Table 4:** List of up-and down-regulated metabolites in kabuli genotypes of chickpea grown under rainfed condition in comparison to irrigated controls.

S. no.	Compound (CAS registry numbers)	Retention time	*p* (Corr) rainfed vs control	Log FC (abs) rainfed vs control
**List of up-regulated metabolites**				
1	Malic acid (38166-11-9)	21.60	7.6E−3	0.89
2	Threonic acid (13752-84-6)	23.58	1.4E−2	0.80
3	Malonic acid (18457-04-0)	13.67	1.4E−2	0.80
4	D-Fructose (19126-98-8)	29.16	3.0E−3	0.99
5	208.0*	54.00	2.2E−3	1.02
**List of down-regulated metabolites**				
1	Trehalose (42390-78-3)	43.90	2.2E−3	− 1.02
2	Sucrose (19159-25-2)	44.85	2.5E−04	− 1.19
3	73.0*	28.79	8.7E−06	− 1.38
4	Mannofuranoside (6737-01-5)	28.10	6.4E−06	− 1.40
5	Arabino-hexos-2-ulose (74685-71-5)	21.80	7.5	− 0.90
6	Maltose (6363-53-7)	43.40	1.10E−07	− 1.55
7	Oxalic acid (18294-04-7)	19.19	3.73E−09	− 1.65
8	β*-d*-Galactofuranoside (55493-81-7)	29.00	8.76E−06	− 1.37

*m/z of base peak of unidentified metabolite.

A prediction model of water-stressed versus control plants was built by using thirteen significantly important metabolites of kabuli genotype of chickpea. PLS-DA score plots showed a clear separation trend between the plants grown under rainfed, and irrigated control conditions (Fig. 2B). Sensitivity and specificity were also measured from the constructed model which was found to be 94.11% and 100% respectively, while the overall accuracy of the model was 97.14%, (Supplementary Material Table S3). On the basis of observed up- and down-regulated metabolites, oxalic acid, threonic acid, d-Fructose, malic acid, and malonic acid were found to be common between the desi and kabuli genotypes.

### Pathway analysis

**Figure 4:**
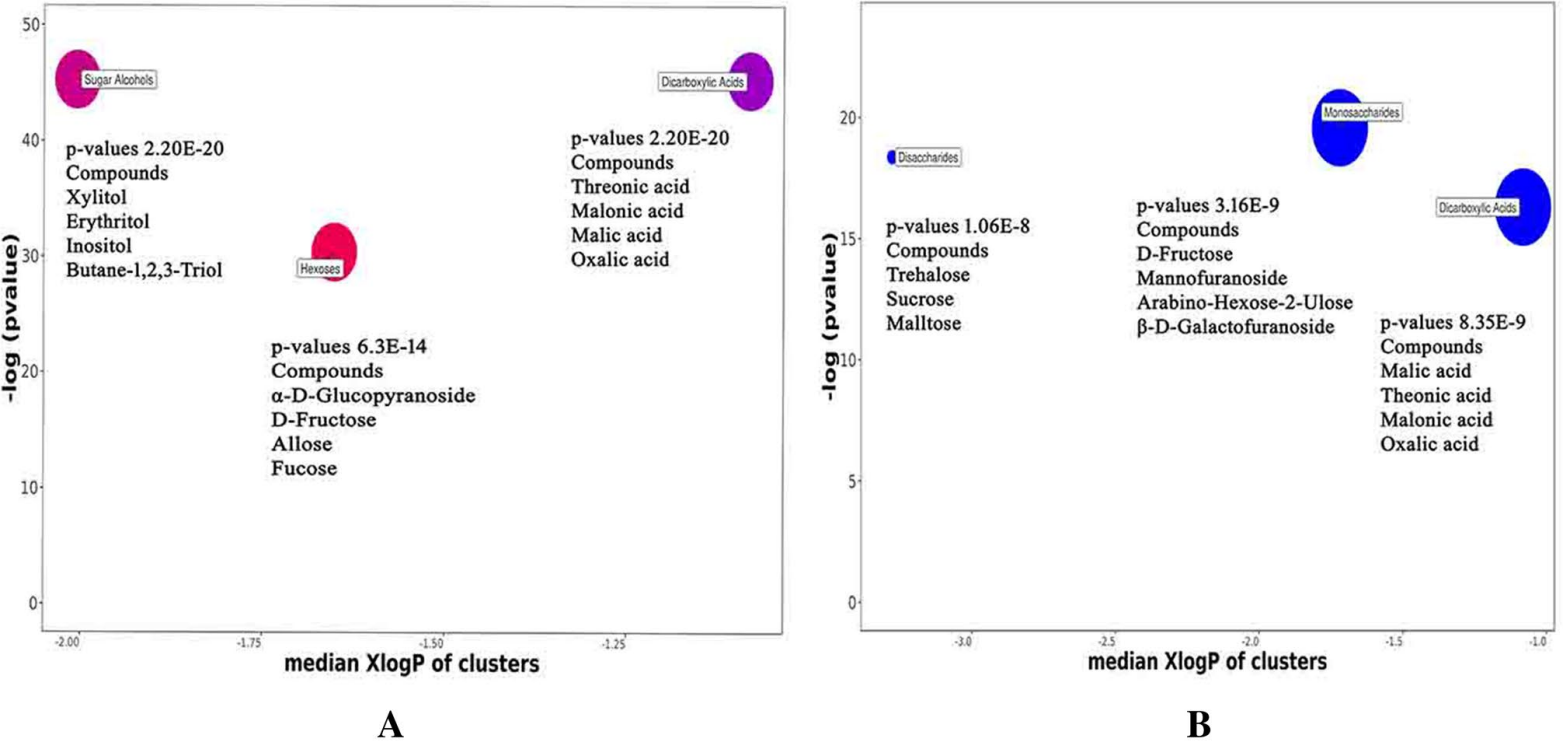
Chemrich enrichment plots showing metabolite clusters with significant impact in chickpea genotypes under rainfed conditions, (**A**) desi genotypes, (**B**) kabuli chickpea genotypes.

In order to find out relevant metabolic pathways using *p*-values and fold change of each metabolite, an online available software ChemRICH was used^18^. Identified metabolites were used to generate the pathways from KEGG metabolic pathway database (Supplementary Material, Figs. S3–S5). In desi chickpea genotypes, three metabolite clusters with significant impact were obtained (Supplementary Material Table S4), including dicarboxylic acid cluster with four altered metabolites; threonic acid, malonic acid, malic acid and oxalic acid. Sugar alcohols cluster with four altered metabolites including xylitol, erythritol, inositol and butane-1,2,3-triol. Hexose cluster including four monosaccharides, α-d-Glucopyranoside, d-fructose, allose and fucose. The key compounds of the above mentioned clusters were threonic acid, xylitol and α-d-Glucopyranoside respectively. (Fig. 4A). From the sugar alcohol cluster inositol was found to be involved in inositol phosphate metabolism which plays an important role in diverse cellular functions, such as cell growth, cell migration, apoptosis, endocytosis, and cell differentiation. As in the desi genotypes, inositol was found to be up-regulated in the plants grown in the rainfed condition as compared to irrigated control plants, this is clearly showing that the inositol phosphate metabolism was perturbed in the rainfaid plants. Because inositol serves as an osmoprotectant, we can state that inositol phosphate metabolism served as a defense strategy in desi chickpea genotypes against limited water stress. From the monosaccharides cluster, D-fructose was found to be involved in the starch and sucrose metabolism.

In kabuli chickpea genotypes, metabolite clusters with significant impact were of monosaccharides including d-fructose, mannofuranoside, Arabino-hexos-2-ulose and β-d-Galactofuranoside. Disaccharides cluster including three compounds; trehalose, sucrose and maltose. Dicarboxylic acid cluster includes four metabolites namely malic acid, malonic acid, threonic acid and oxalic acid. The key compounds of these clusters were mannofuranoside, maltose, and oxalic acid respectively (Fig. 4B). From sugar clusters of monosaccharides and disaccharides d-fructose, trehalose, sucrose, and maltose were found to be involved in starch and sucrose metabolism. Sucrose is the end product of photosynthesis, serves in the production of energy, and the synthesis of complex carbohydrate. Among the 4 metabolites involved in starch and sucrose metabolism, d-fructose also indirectly influenced the amino sugar and nucleotide sugar metabolism.

## Discussion

In this study, we have identified 19 significant metabolites from the desi chickpea samples and 13 from the kabuli chickpea samples. The identified metabolites include sugars, sugar alcohols, organic acids, alcohols, and amino acids. A recent study on chickpea plants grown in pots under drought stress has identified mainly amino acids^14^. They have used the controlled green house environment for inducing drought effects on plants and analyzed samples with a modified C18 column (PFP). However, we have selected field grown chickpea plant samples which could provide the metabolic changes induced by the limited water availability as well as other environment and field effects, and samples were analyzed through a GC–MS based untargeted metabolomics approach.

An up-regulation of sugars such as D-fructose, fucose and α D-glucpyranoside was observed in desi genotype of chickpea grown under the rainfed condition in comparison with their irrigated control plants. Sugars are among the compatible solutes that function as osmoprotectants in plants and help them to survive in extreme environmental conditions^19^. During water stress, they protect the plants from degradation by reducing the effects of osmotic stress resulting from the shortage of water, maintaining the turgidity of leaves and prevent proteins from dehydration. Sugar accumulation prevents cell membrane from oxidation under water deficiency^20^ and decreases the rate of photosynthesis in the plants grown under limited water conditions. An up-regulation of α-d-glucopyranoside, which is a derivative of glucose, was observed, while accumulation of glucose in plants under limited water condition induces stomatal closure, and impose plant adaptability under drought stress^19,21^. An up-regulation of allose sugar which belongs to the group of rare monosaccharides, was also observed in desi chickpea genotypes^22^.

Up-regulation of l-proline was also observed in desi chickpea genotypes grown in the rainfed condition as compared to the irrigated control. According to previous studies proline accumulates in significant quantities in plants under several biotic and abiotic stresses including water stress^11^. It plays an essential role in plants grown under drought stress conditions. For example, proline reduces the oxidative damages of membrane caused by reactive oxygen species, and improves signal transduction pathways. It also stabilizes DNA and protein complexes^23^. Up-regulation of l-proline in desi chickpea plants grown under the rainfed condition as compared to the irrigated plants in this study, suggest that desi chickpea genotypes has accumulated proline for the maintenance of cellular homeostasis, and compensating the adverse effects coming from the limited availability of water. Sugar alcohols, polyols, inositol and myo-inositol were up-regulated in desi genotypes of chickpea grown in rainfed condition. Polyols are involved in stabilizing the macromolecules and scavenging hydroxyl radicals and in this way preventing membranes and enzymes from oxidative damage. Accumulation of polyols in plants is directly related to their tolerance to water stress. They function either by osmotic adjustment, facilitating the retention of water in cytoplasm, and allowing sodium sequestration to the vacuole or apoplast. This might also function through protection of cellular structure by scavenging reactive oxygen species^24,25^. Malic acid, butane-1,2,3-triol, glyceric acid, and ethylamine were also found to be up-regulated. Malic acid accumulation may not be because of the drought stress but rather due to the stomatal regulatory system that works in tandem with a plant hormone abscisic acid^26^. However, down-regulation of threonic acid, malonic acid, oxalic acid, gluconic acid, xylitol, erythritol, and arabinofuranose was observed.

The data of kabuli chickpea genotypes showed a down-regulation of six sugars, trehalose, arabino-hexose-2-ulose, maltose, sucrose, mannofuranoside, and α-d-glactofuranoside as well as oxalic acid, while up-regulation of d-fructose and three acids; malic acid, threonic acid, and malonic acid in the rainfed kabuli chickpea genotypes as compared to their irrigated control. Sucrose is the most frequently reported sugar in stress response. Its accumulation is normally associated with higher stress tolerance^27^. Down-regulation of the sugars in rainfed samples in comparison to irrigated control samples suggests that these plants were unable to accumulate osmoprotectants and protect themselves from adverse effects of limited water supply. The metabolic rate of citric acid cycle is also affected by limited water supply. Plants decrease stomatal conductance during water scarcity, it has been noted that the mitochondrial respiration is reduced under water stress conditions due to reduced carbohydrate synthesis resulting from the decrease in CO_2_ fixation. Therefore, because of substrate limitation, it is possible that the intermediates of the citric acid cycle, for example, succinic acid and malic acid exhibit a decreased level under stress conditions^28,29^.

## Conclusion

This study identified differentiating metabolic behavior of chickpea genotypes grown under rainfed conditions versus the same genotypes grown in irrigated conditions as a control in the field. In general, desi type of chickpea was found to be tolerant than kabuli type, because the desi genotype has shown the lesser yield reduction than kabuli chickpea genotype when grown in the field under limited water stress. In agreement with the field data, desi chickpea accumulated compatible solutes as osmoprotectants, such as, myo-inositol, l-proline, d-fructose, allose and fucose. Kabuli genotype of chickpea showed drought intolerant behavior as compatible solutes were down-regulated in rainfed conditions. This indicates that genotypes in this group may give better performance in irrigated conditions. Identified metabolic pathways were found to be important in rainfed condition and most probably compensating the adverse effects of limited water stress. It is expected that the findings of this study will help to understand the biochemical status of chickpea plants under limited water stress and can be used to monitor and assess performance in crop breeding programs.

## Materials and methods

### Cultivation of chickpea and sample collection

A total of forty four advanced breeding lines of chickpea were used in this study which comprised 18 kabuli and 26 desi genotypes. Seeds of these genotypes were sown in the field at NIAB, Faisalabad, as irrigated control, Water was supplied before sowing and no water was supplied through irrigation up to maturity to avoid excessive vegetative growth. Chickpea produces more grains under limited water conditions because with excessive water or irrigation chickpea plants begin producing more leaves than pods. Another set of forty four genotypes was sown in the field under rainfed conditions at Kaloor Kot during the season of 2016–2017 (Supplementary Information Table S1). The experiment was conducted in three replicates with a randomized complete block (RCBD) design having row length 3 m, row to row distance of 30 cm and plant to plant distance of 15 cm for each genotype. The soil moisture content, measured randomly at the time of sampling, was 15–17% at Kalur kot and 25–30% at NIAB. Leaf samples were collected at the time of pod setting from the middle part of the plant (Supplementary Material, Fig. 2 for metabolite analysis.

### Chemicals and reagents

All the reagents used during the study were of analytical grade. Methanol and pyridine were purchased from Tedia (Tedia way, Fairfield, USA). MSTFA (*N*-methyl-*N* (trimethylsilyl) trifluoroacetamide) was purchased from Chem-Impex International Inc. (Wood Dale, Illinois, USA). Methoxyamine hydrochloride and ribitol were purchased from Sigma Aldrich (St. Louis, MO, USA). Deionized water (Milli-Q) was used throughout the study (Millipore, Billerica, MA, USA).

### Sample preparation and derivatization

Leaf samples were ground to a powder by using standard procedure^30^. And all three biological replicates for the individual genotypes were pooled for GC–MS analysis. Enzyme inactivation was done by the addition of 700 μL methanol (precooled at – 20 °C) in 150 mg of plant sample containing 60 μL of ribitol (0.4 mg/mL stock in milli-Q water) as an internal quantitative standard in a 2 mL Eppendorf tube. The tube was vortexed for 10 s, and then shaken on the thermomixer at 800 rpm for 15 min at 70 °C. After mixing the tube was centrifuged at 11,000×*g* for 15 min, the supernatants were transferred into another 2 mL Eppendorf tube and 700 μL milli-Q water and 370 μL chloroform were added. The tube was vortexed and centrifuged at 2,200×*g* for 10 min^30^. Aliquots of polar layer (150 μL) were dried in a vacuum concentrator. The dried extract was stored at − 20 °C until the time of analysis. The dried samples were then derivatized with the addition of 40 μL methoxyamine hydrochloride in pyridine (20 mg/mL). The tube was vortexed and mixed on the thermomixer at 30 °C for a 1.5 h and then 70 μL MSTFA was added to convert the organic acids into volatile trimethylsilyl derivative, and mixed for 30 min at 30 °C.

### GC–MS analysis

GC–MS analysis was performed on 7890A gas chromatography (Agilent Technologies, USA), equipped with an Agilent Technology GC autosampler 120 (PAL-LHX-AG12), coupled to an Agilent 7000 Triple Quad system (Agilent Technologies, USA). HP-5MS 30 m and 0.250 (mm) diameter fused silica capillary column (Agilent J&W Scientific, Folsom, CA, USA), chemically bonded with a 5% diphenyl and 95% dimethylpolysiloxane cross-linked stationary phase (0.25 mm film thickness) was used. The injection volume for GC–MS analysis was 1 μL of the derivatized sample extract. Helium was used as a carrier gas with a flow rate of 1 mL/min. The sample was injected in split mode. Injection temperature was 230 °C. The oven temperature was kept isothermal at 70 °C for 5 min, followed by 5 °C per min ramp to 310 °C. Ribitol was used as a quality control standard before and after every batch, and the mean value obtained from its retention time was 27.32 min with standard deviation of 0.02. A blank was run between the samples in order to remove the contamination. Electron ionization was used as an ionization source for GC–MS analysis at 70 eV. Data was acquired in the full scan mode from *m/z* 50 to 650 with a scan time of 0.5 s. Perfluorotributylamine (PFTBA) was used for mass calibration.

### GC–MS data processing and statistical analysis

Data processing was performed by Agilent Mass Hunter Qualitative Analysis (version B.04.00). In this study, peak integration parameters on Mass Hunter were set as previously reported^31,32^. MSI level 2 identification of GC–MS peaks was carried out by comparing the mass spectra with already existing data in the NIST mass spectral (Wiley registry) library. The spectral peak matching was set at ≥ 70% similarity index for metabolite identification. All the GC–MS spectra with identification information was converted to CEF format, and exported to MPP (Mass Profile Professional) for further processing. The data was then filtered at the minimum absolute abundance of 5,000 counts, and with minimum 3 number of ions. Alignment parameters were set as retention time tolerance 0.05 min, match factor 0.3, and Delta MZ (low resolution) 0.2. External scalar was used to normalize the data. Z-transform was selected as a baseline option treating all the compounds equally regardless of their intensities. After alignment of the data and compound detection, the compounds obtained were filtered by frequency, fold change, and *p*-value. Principle Component Analysis (PCA) models and bar charts of each group were made for the comparison of highly expressed metabolites in rainfed and irrigated conditions.

## Supplementary information

Supplementary Information.

## References

[1] Heidarvand L, Maali-Amiri R (2013). Physio-biochemical and proteome analysis of chickpea in early phases of cold stress. J. Plant Physiol..

[2] Rao, P. P., Birthal, P., Bhagavatula, S. & Bantilan, M. Chickpea and pigeonpea economies in Asia: facts, trends and outlook. (2010).

[3] Rachwa-Rosiak D, Nebesny E, Budryn G (2015). Chickpeas-composition, nutritional value, health benefits, application to bread and snacks: a review. Crit. Rev. Food Sci. Nutr..

[4] Cobos MJ (2016). Genotype and environment effects on sensory, nutritional, and physical traits in chickpea (*Cicer arietinum *L.). Spanish J. Agric. Res..

[5] Maheri-Sis N, Chamani M, Ali-Asghar S, Mirza-Aghazadeh A, Aghajanzadeh-Golshani A (2008). Nutritional evaluation of kabuli and desi type chickpeas (*Cicer arietinum* L.) for ruminants using in vitro gas production technique. Afr. J. Biotechnol..

[6] Jukanti AK, Gaur PM, Gowda CL, Chibbar RN (2012). Nutritional quality and health benefits of chickpea (*Cicer arietinum* L.): a review. Br. J. Nutr..

[7] Krishnamurthy L, Ito O, Johansen C, Saxena NP (1998). Length to weight ratio of chickpea roots under progressively receding soil moisture conditions in a Vertisol. Field Crops Res..

[8] Pang J (2017). Response of chickpea (*Cicer arietinum* L.) to terminal drought: leaf stomatal conductance, pod abscisic acid concentration, and seed set. J. Exp. Bot..

[9] Awasthi R (2014). Individual and combined effects of transient drought and heat stress on carbon assimilation and seed filling in chickpea. Funct. Plant Biol..

[10] Todaka D (2017). Temporal and spatial changes in gene expression, metabolite accumulation and phytohormone content in rice seedlings grown under drought stress conditions. Plant J..

[11] AlHassan M, Fuertes MM, Sánchez FJR, Vicente O, Boscaiu M (2015). Effects of salt and water stress on plant growth and on accumulation of osmolytes and antioxidant compounds in cherry tomato. Notulae Bot. Horti Agrobot. Cluj-Napoca.

[12] Feussner I, Polle A (2015). What the transcriptome does not tell: proteomics and metabolomics are closer to the plants' patho-phenotype. Curr. Opin. Plant Biol..

[13] Mafakheri A, Siosemardeh A, Bahramnejad B, Struik P, Sohrabi Y (2010). Effect of drought stress on yield, proline and chlorophyll contents in three chickpea cultivars. Aust. J. Crop Sci..

[14] Khan N, Bano A, Rahman MA, Rathinasabapathi B, Babar MA (2018). UPLC-HRMS-based untargeted metabolic profiling reveals changes in chickpea (*Cicer arietinum*) metabolome following long-term drought stress. Plant Cell Environ..

[15] Farooq M, Ullah A, Lee D-J, Alghamdi SS (2018). Terminal drought-priming improves the drought tolerance in desi and kabuli chickpea. Int. J. Agric. Biol..

[16] Yadav SS (2007). Evaluation of Helicoverpa and drought resistance in desi and kabuli chickpea. Plant Genet. Resour..

[17] Farooq M, Ullah A, Lee DJ, Alghamdi SS, Siddique KHM (2018). Desi chickpea genotypes tolerate drought stress better than kabuli types by modulating germination metabolism, trehalose accumulation, and carbon assimilation. Plant Physiol. Biochem..

[18] Barupal DK, Fiehn O (2017). Chemical Similarity Enrichment Analysis (ChemRICH) as alternative to biochemical pathway mapping for metabolomic datasets. Sci. Rep..

[19] Sami F, Yusuf M, Faizan M, Faraz A, Hayat S (2016). Role of sugars under abiotic stress. Plant Physiol. Biochem..

[20] Arabzadeh N (2012). The effect of drought stress on soluble carbohydrates (sugars) in two species of *Haloxylon persicum* and *Haloxylon aphyllum*. Asian J. Plant Sci..

[21] Osakabe Y, Yamaguchi-Shinozaki K, Shinozaki K, Tran LS (2014). ABA control of plant macroelement membrane transport systems in response to water deficit and high salinity. New Phytol..

[22] Kano A (2013). The rare sugar D-allose acts as a triggering molecule of rice defence via ROS generation. J. Exp. Bot..

[23] Yaish MW (2015). Proline accumulation is a general response to abiotic stress in the date palm tree (*Phoenix dactylifera* L.). Genet. Mol. Res..

[24] Dumschott K, Richter A, Loescher W, Merchant A (2017). Post photosynthetic carbon partitioning to sugar alcohols and consequences for plant growth. Phytochemistry.

[25] Gupta AK, Kaur N (2005). Sugar signalling and gene expression in relation to carbohydrate metabolism under abiotic stresses in plants. J. Biosci..

[26] Wilkinson S, Davies WJ (2002). ABA-based chemical signalling: the co-ordination of responses to stress in plants. Plant Cell Environ..

[27] Jorge TF (2017). GC-TOF-MS analysis reveals salt stress-responsive primary metabolites in *Casuarina glauca* tissues. Metabolomics.

[28] Barchet GL (2014). Investigating the drought-stress response of hybrid poplar genotypes by metabolite profiling. Tree Physiol..

[29] Lemoine R (2013). Source-to-sink transport of sugar and regulation by environmental factors. Front. Plant Sci..

[30] Lisec J, Schauer N, Kopka J, Willmitzer L, Fernie AR (2006). Gas chromatography mass spectrometry-based metabolite profiling in plants. Nat. Protoc..

[31] Musharraf SG, Mazhar S, Choudhary MI, Rizi N, Attaur R (2015). Plasma metabolite profiling and chemometric analyses of lung cancer along with three controls through gas chromatography-mass spectrometry. Sci. Rep..

[32] Musharraf SG, Mazhar S, Siddiqui AJ, Choudhary MI, Atta ur R (2013). Metabolite profiling of human plasma by different extraction methods through gas chromatography-mass spectrometry: an objective comparison. Anal. Chim. Acta.

