# Supplementary material for: A comparative metabolomic study on desi and kabuli chickpea (Cicer arietinum L.) genotypes under rainfed and irrigated field conditions
Source: Sci Rep. 2020 Aug 18;10:13919. doi: 10.1038/s41598-020-70963-6 (PMC7434909; doi:10.1038/s41598-020-70963-6)
Supplement: Supplementary file 1 — Supplementary Information. [file 41598_2020_70963_MOESM1_ESM.pdf]

Supplementary Material

A Comparative Metabolomic Study on Desi and Kabuli Chickpea (*Cicer arietinum* L.) genotypes under Rainfed and Irrigated Field Conditions

Zaib Un Nisa[a], Anjuman Arif[c], Muhammad Qandeel Waheed[c], Tariq Mahmood Shah[c], Ayesha Iqbal[b], Amna Jabbar Siddiqui[b], Muhammad Iqbal Choudhary[a,b,d], Hesham R. El-Seedi[e,f], Syed Ghulam Musharraf[a,b*]

[a] *H.E.J. Research Institute of Chemistry, International Center for Chemical and Biological Sciences, University of Karachi, Karachi-75270, Pakistan*
[b] *Dr. Panjwani Center for Molecular Medicine and Drug Research, International Center for Chemical and Biological Sciences, University of Karachi, Karachi-75270, Pakistan*
[c] *Nuclear Institute for Agriculture & Biology (NIAB), Faisalabad-38000, Pakistan*
[d] *Department of Biochemistry, Faculty of Science, King Abdulaziz University, Jeddah-21589, Saudi Arabia*
[e] *Pharmacognosoy Group, Department of Medicinal Chemistry, Uppsala University, Biomedical Centre, Box 574, 75 123 Uppsala, Sweden*
[f] *Alrayan Medical College, Medina 42541, Kingdom of Saudi Arabia*

* Corresponding author. Tel.: +92 21 34824924-5; 34819010; fax: + 92 21 34819018-9.
E-mail address: musharraf1977@yahoo.com

Table S1: Environment description of control and drought experiments during October 2016 to March 2017.

Experiment	Environment	October	November	December	January	February	March	Average
**Control (Irrigated):**								
Faisalabad	**Temp (min)**	23	17	13	10	13	17	15
Latitude: 31.450	**Temp (max)**	37	29	24	24	26	32	29
Longitude: 73.135	**Sunny days**	31	28	31	31	25	22	28
Soil type:	**Rain fall**	0	0	0	13	84	32	22
Silt loam or very fine sandy loam*	**Humidity**	17	23	24	26	35	33	26
**Drought (Rainfed):**								
Kallur kot	**Temp (min)**	25	17	13	11	13	20	16
Latitude: 32.156	**Temp (max)**	36	27	23	22	24	31	27
Longitude: 71.272	**Sunny days**	31	29	30	31	22	20	27
Soil type:	**Rain fall**	0	2	0	0	5	9	2
Sandy loam**	**Humidity**	15	23	21	22	34	34	25

Temperature is measured in °C (minimum and maximum), average rainfall in mm and humidity in %.

*Silt soils are fertile, light but moisture-retentive and easily compacted.

**Sandy loams are capable of quickly draining excess water and usually deficient in micronutrients, especially zinc and iron.

Table S2: Confusion matrix of model generated on data of rainfed and control desi type of plants (26 lines).

	Control Predicted	Rainfed predicted	Accuracy
True control	26	0	100.00
True drought	0	25	100.00
Overall Accuracy			100.00

Table S3: Confusion matrix of model generated on data of rainfed and control kabuli type of plants (18 lines).

	Control Predicted	Rainfed predicted	Accuracy
True control	16	1	94.11
True drought	0	18	100.00
Overall Accuracy			97.14

Table S4. List of altered metabolites in desi and kabuli groups of chickpea plant samples under rainfed condition *versus* irrigated control.

**Cluster name**	**Cluster size**	**p-values**	**FDR**	**Key compound**	**Altered metabolites**	**Increased**	**Decreased**	**Increased ratio**	**Altered Ratio**
**Cluster level enrichment result of desi chickpea genotype**									
Dicarboxylic Acids	4	2.2E-20	3.3E-20	Threonic acid	4	1	3	0.2	1
Sugar Alcohols	4	2.2E-20	3.3E-20	Xylitol	4	2	2	0.5	1
Hexoses	4	6.3E-14	6.3E-14	α-D-Glucopyranoside	4	3	1	0.8	1
**Cluster level enrichment result of kabuli chickpea genotypes**									
Monosaccharides	4	3.2E-09	9.5E-09	Mannofuranoside	4	0	4	0	1
Disaccharides	3	1.10E-08	1.60E-08	Maltose	3	0	3	0	1
Dicarboxylic Acids	4	8.40E-08	8.40E-08	Oxalic acid	4	0	4	0	1

Figure S1: Box plot of mean (A) and individual value (B) of chickpea genotypes in non-stressed (control) and stressed (drought) conditions

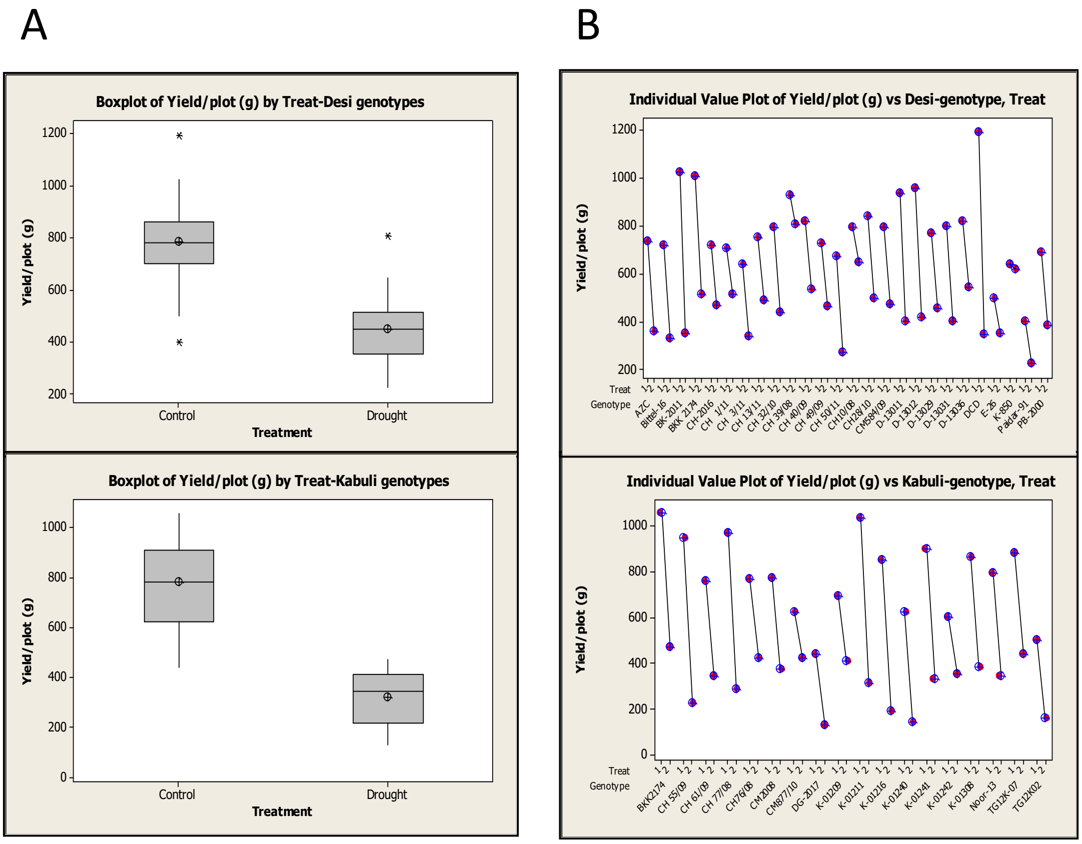

Figure S2: A diagramatic representation of sample collection for metabolite analysis

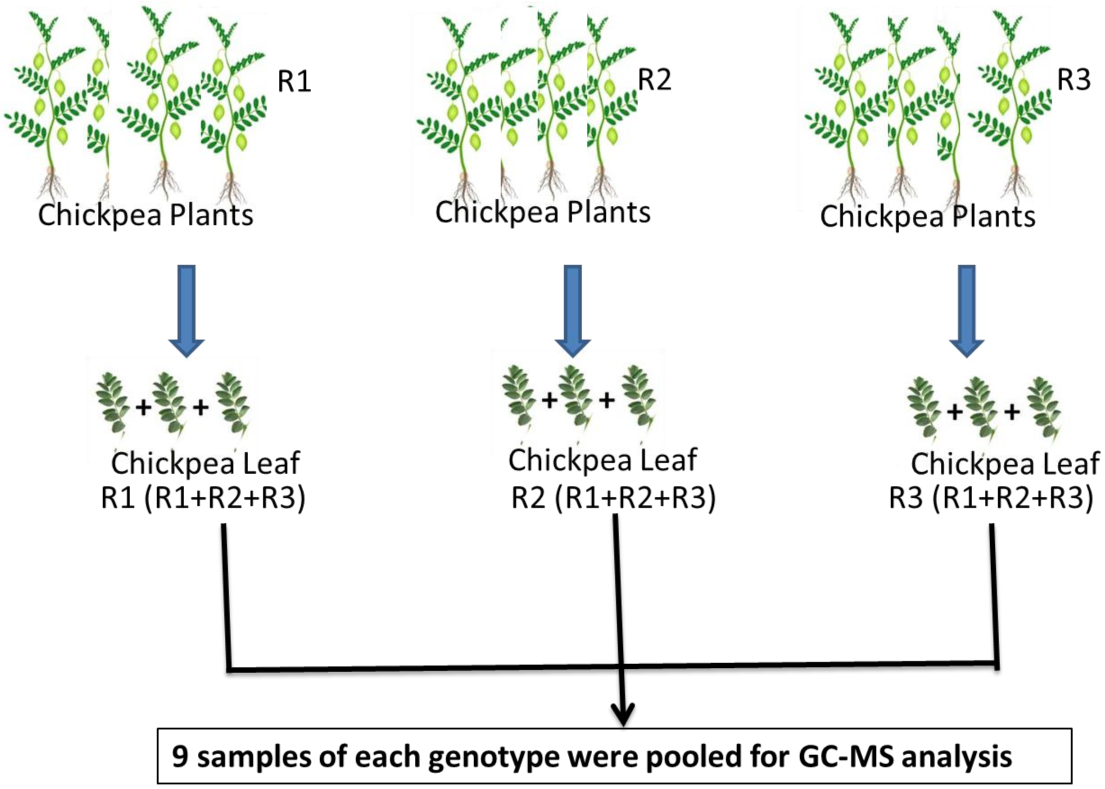

Figure S3: Pathways generated by list of metabolites discriminating between irrigated controls and drought stressed samples of chickpea plants.

Starch and sucrose metabolism [1]

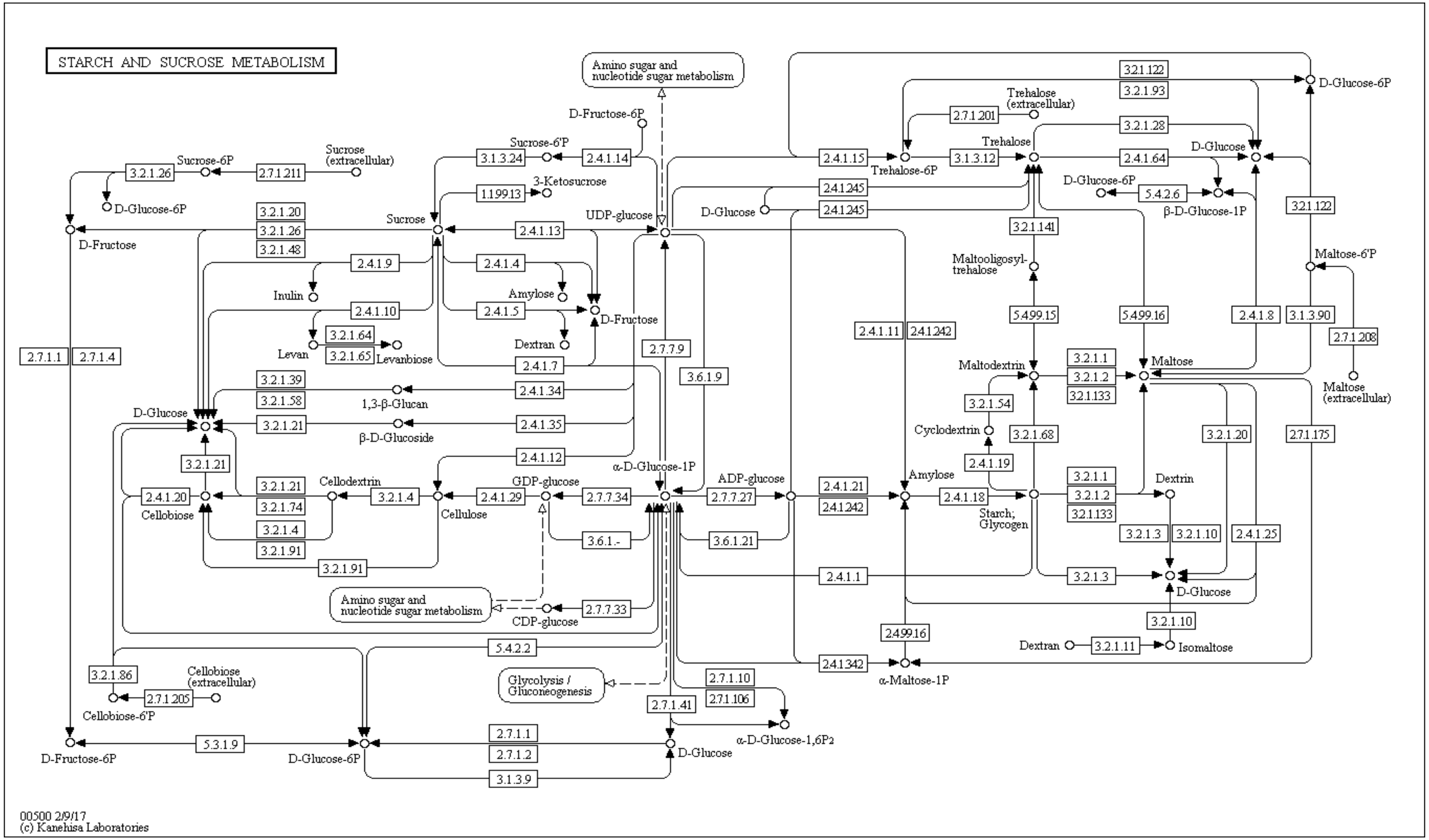

Figure S4: Citrate cycle (TCA cycle) [1].

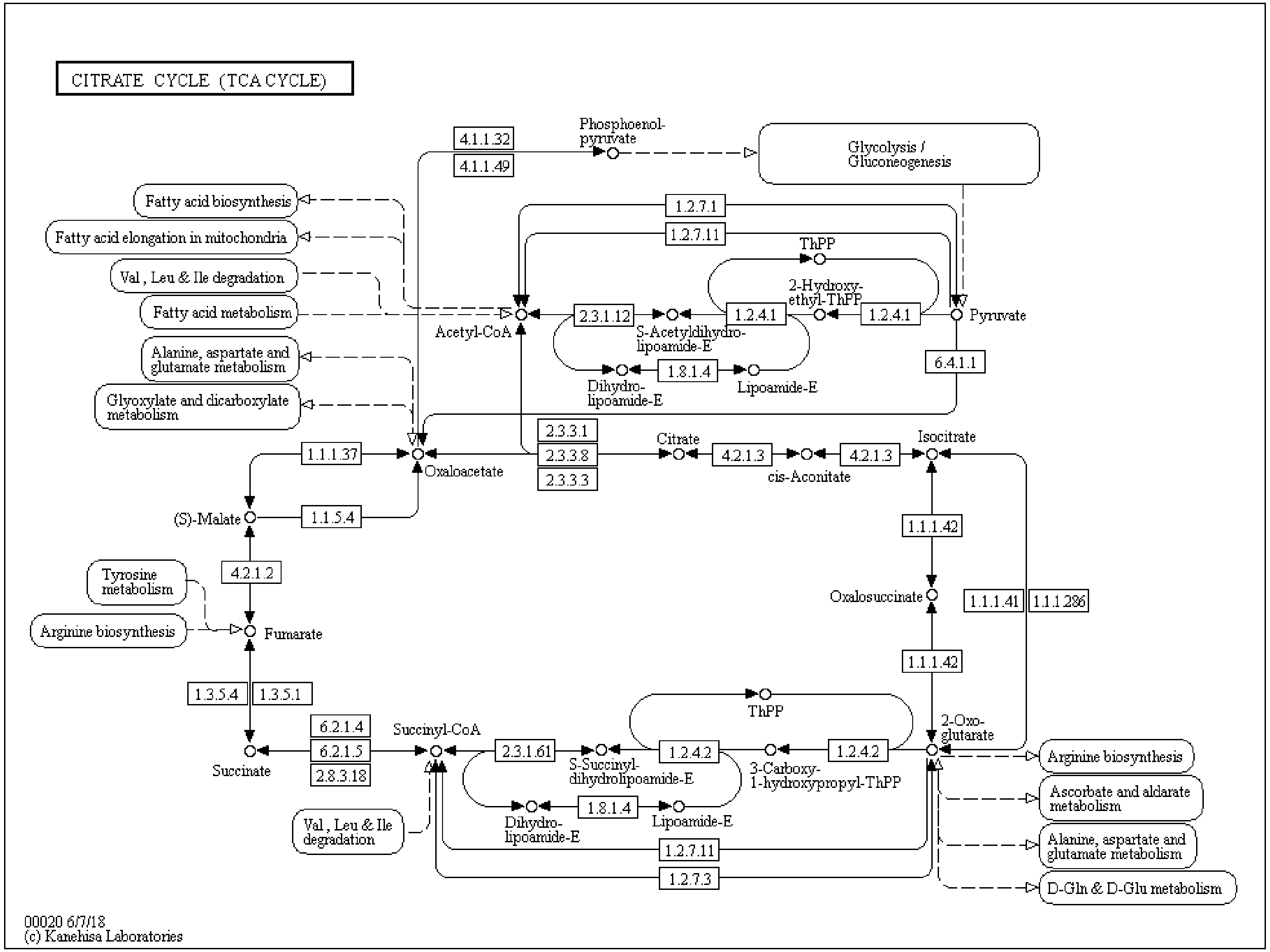

Figure S5: Inositol Phosphate Metabolism [1]

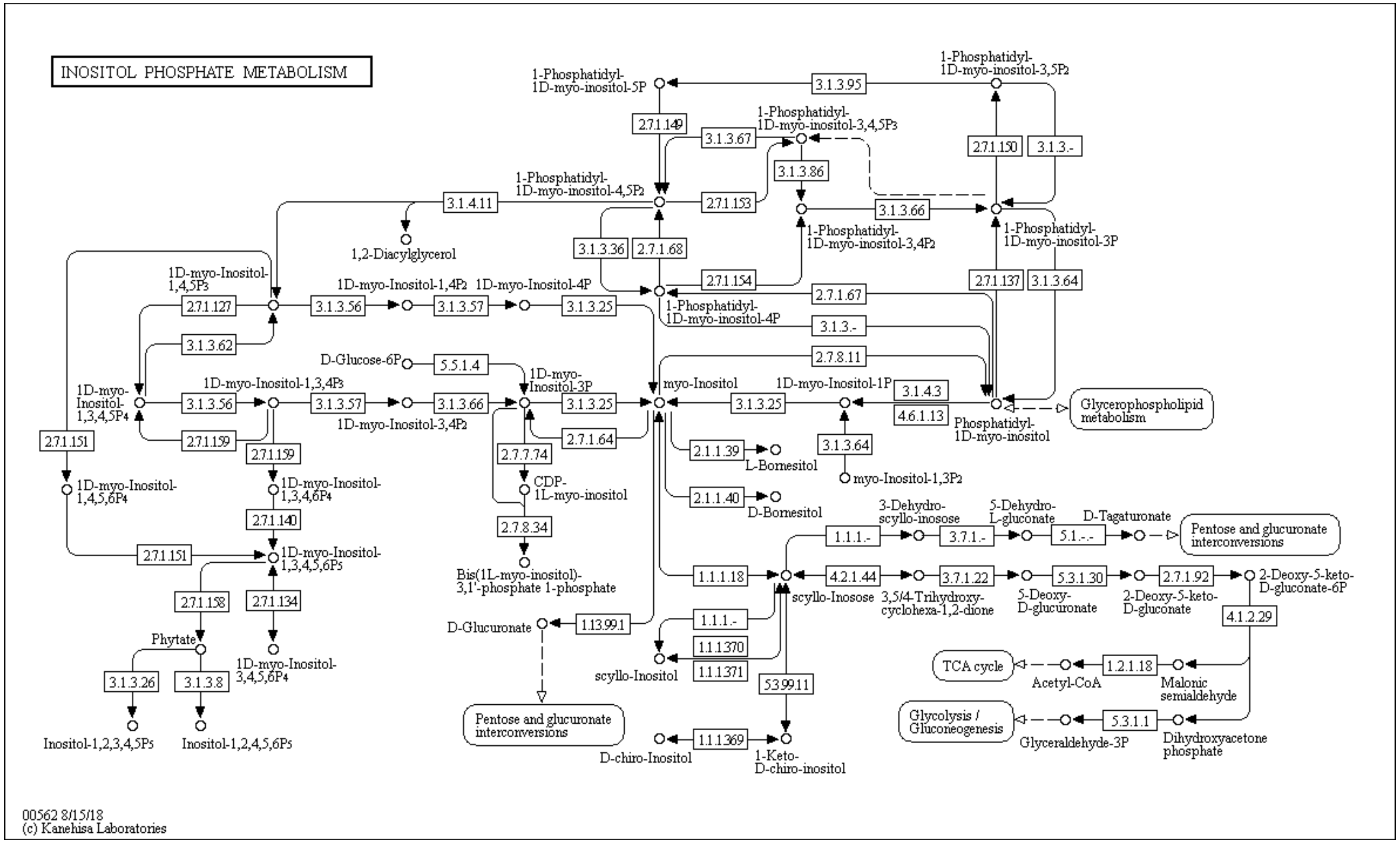

Reference

[1] Kanehisa M, Furumichi M, Tanabe M, Sato Y, Morishima K. KEGG: new perspectives on genomes, pathways, diseases and drugs. Nucleic Acids Res 2017;45:D353-D61.
